# The Effects of Trabeculectomy on Pseudoexfoliation Glaucoma and Primary Open-Angle Glaucoma

**DOI:** 10.1155/2020/1723691

**Published:** 2020-03-23

**Authors:** Fan Li, Guangxian Tang, Hengli Zhang, Xiaowei Yan, Lihua Ma, Yulei Geng

**Affiliations:** Department of Ophthalmology, Shijiazhuang No. 1 Hospital, Hebei 050000, China

## Abstract

**Purpose:**

To compare long-term effects of trabeculectomy on pseudoexfoliation glaucoma (PXG) and primary open-angle glaucoma (POAG).

**Methods:**

This retrospective case-control study included 53 eyes of PXG and 76 eyes of POAG. Intraocular pressure (IOP), number of antiglaucoma medications used, surgical success rate, and occurrence of complications were observed and statistically analyzed in both groups at 3 and 6 months and at 1, 3, and 5 years after trabeculectomy. Surgical success was defined according to the following 3 criteria: (1) IOP ≤ 21 mmHg; (2) IOP ≤ 18 mmHg; (3) IOP ≤ 15 mmHg. Complete success is defined as patients met these criteria without medical treatment, and qualified success is defined as patients met these criteria with medical treatment (≤3 medications). Cumulative probabilities of success were compared using the Kaplan–Meier survival analysis.

**Results:**

For the 3 criteria, there were no statistically significant differences in complete and qualified success rates between the two groups at 3 and 6 months after trabeculectomy (*P* > 0.05). For criterion A, complete success rates in PXG at 3 and 5 years after surgery were lower than those in POAG; for criterion B, complete and qualified success rates in PXG at 3 and 5 years after surgery were lower than those in POAG; for criterion C, complete and qualified success rates in PXG at 1, 3, and 5 years after surgery were lower than those in POAG, the differences were statistically significant (*P* < 0.05).

**Conclusions:**

The short-term success rates of both types of glaucoma were similar; however, the long-term success rate of PXG was significantly lower, and it was difficult to achieve long-term control of IOP at a low target level.

## 1. Introduction

Pseudoexfoliation glaucoma (PXG) is a common type of secondary glaucoma caused by pseudoexfoliation syndrome (PEX). Compared with primary open-angle glaucoma (POAG), PXG is characterized by high mean and peak intraocular pressure (IOP), a wide fluctuation range, more severe visual field damage, and faster progression [1–5]. PXG exhibits poor response to glaucoma medications, which usually cannot control its progression [6]; thus, antiglaucoma surgical treatment—most commonly trabeculectomy—is required [7]. Although significant progress has been made recently in surgical techniques and postoperative care, excessive scarring and tissue fibrosis caused by proliferation of conjunctival fibroblasts and deposition of extracellular matrix after trabeculectomy are the primary obstacles affecting the formation of filtering blebs and reduction of IOP [8, 9]. This study aimed to evaluate the clinical effects of trabeculectomy on the treatment of PXG and POAG, to calculate the long-term success rates of the surgery in both types of glaucoma, and to explore risk factors affecting the success of the surgery.

## 2. Materials and Methods

### 2.1. Patients

Continuous selection of fifty-three PXG patients (53 eyes), who underwent trabeculectomy in the Glaucoma Department of Shijiazhuang No. 1 Hospital between January 2014 and June 2019, comprised the PXG group. Seventy-six sex-, age-, and axial length-matched patients with POAG (76 eyes) comprised the control POAG group. All patients underwent trabeculectomy for IOP reduction. This study complied with the Declaration of Helsinki and was approved by the Ethics Committee of Shijiazhuang No. 1 Hospital. All patients provided written informed consent. There was no significant difference in age, sex, axial length, preoperative IOP, antiglaucoma medications, or mean defect (MD) between the two groups (*P* > 0.05) (Table 1).

### 2.2. Inclusion and Exclusion Criteria

#### 2.2.1. Inclusion Criteria

Eyes in the PXG group met the following PXG diagnostic criteria: gray-white exfoliating materials were found at the pupillary margin, iris surface, and anterior lens capsule; the IOP was >21 mmHg; glaucomatous optic neuropathy and visual field changes [10]. The surgical indications of the PXG group are based on target IOP and benefit-to-risk ratio for individual glaucoma patients. Those with a definite diagnosis of visual field defect and optic nerve damage, for whom the maximum drug tolerance (3 medications) still could not control IOP, those with poor response to glaucoma medications, those who refused drug therapy, or those who experienced drug side effects and systemic contraindications were ruled out.

#### 2.2.2. Exclusion Criteria

Individuals who had undergone glaucoma filtration surgery or other eye surgeries, those with angle-closure glaucoma, optic nerve diseases, or retinal diseases, such as age-related macular degeneration, hypertensive retinopathy, diabetic retinopathy, and retinal vein occlusion, which could affect the results of this study, glaucoma patients with absolute glaucoma or ocular prosthesis in one eye, and those who could not comply with examinations or regular follow-ups were excluded from the study.

### 2.3. Surgical Procedure

A conjunctival flap was made with the fornix as the base, a rectangular scleral flap, 4 mm × 3 mm in size, was made with the upper corneal margin as the base,; and cotton sheets soaked with mitomycin C solution (0.2–0.4 mg/mL) were placed under the scleral and conjunctival flaps for 2–5 min. Balanced salt solution (60 ml) was used for flushing. Corneoscleral trabecular tissue, 3 mm × 1.5 mm, was removed, and a wide-base peripheral iridectomy was performed. The two posterior corners of the scleral flap were sutured using a 10/0 nylon thread under moderate tension. A pair of external removable sutures with relatively high tension was placed on the vertical side incisions on both sides of the scleral flap to achieve relatively firm temporary suture of the scleral flap. The anterior chamber was reconstructed by injecting balanced salt solution. The bulbar conjunctival tissue was sutured using a 10/0 nylon thread. Antibiotics and corticosteroid eye drops were applied topically for 4 to 6 weeks after the surgery, and sutures were removed at the appropriate time. All surgeries were performed by the same senior surgeon. During follow-up, removal of the releasable suture or laser suture lysis was performed if inadequate filtering bleb was noted. Increased IOP despite medical treatment and requirements for a second glaucoma surgery were considered as “end of the follow-up period.”

### 2.4. Observation Indicators

#### 2.4.1. Routine Examinations

All patients underwent systematic ophthalmic examinations, including visual acuity, slit-lamp microscopy, IOP (Goldmann applanation tonometry), gonioscopy, fundus examination, axial length examination, and visual field examination. IOP, number of antiglaucoma medications, complications, and surgical success rate were observed and recorded before surgery at 3 and 6 months and at 1, 3, and 5 years after surgery.

#### 2.4.2. Visual Field Examination

A Humphrey 750i Visual Field Analyzer (Carl Zeiss, Germany) was used for visual field examination in all subjects. The SITA-Fast 30-2 testing procedure was adopted. The reliability criteria were as follows: fixation loss rate <20%, false-negative rate <15%, and false-positive rate <15%. Those who did not meet the criteria were excluded.

### 2.5. Surgical Success Rates

Complete surgical success was defined as postoperative IOP ≤21 mmHg according to criterion A, postoperative IOP ≤18 mmHg according to criterion B, postoperative IOP ≤15 mmHg according to criterion C, and no use of topical antiglaucoma medications for all criteria. Qualified success was defined as the use of ≤3 antiglaucoma medications after surgery and fulfillment of the above three IOP control criteria. Failure to control IOP after surgery was defined as a postoperative IOP higher than the upper limit of the abovementioned IOP criteria, an IOP <6 mmHg, difficulty in controlling IOP with medications or secondary antiglaucoma surgery, or requirement for other IOP reduction treatments.

### 2.6. Statistical Analysis

SPSS version 19.0 (IBM Corporation, Armonk, NY, USA) was used for data analysis. Intragroup comparisons of IOP before and after surgery were performed using the paired *t*-test, while pair-wise comparison between the groups was performed using the independent sample *t*-test. Age, axial length, number of antiglaucoma medications, follow-up period, and MD value of visual field were compared between the two groups using the independent sample *t*-test. The sex composition ratio and complications were compared using the chi-squared test or the Fisher exact test. Cumulative probabilities of success were assessed using Kaplan–Meier survival curves, and the log-rank test was used for group comparisons. Differences with *P* < 0.05 were considered to be statistically significant.

## 3. Results

### 3.1. IOP and Medications

Preoperative and postoperative IOP values of types of glaucoma are summarized in Table 2. In the PXG group, the mean IOP decreased from 28 ± 9 mmHg before surgery (53 eyes) to 17 ± 4 mmHg 5 years after surgery (35 eyes) (*t* = 6.161; *P* < 0.001). The mean IOP in the POAG group decreased from 26 ± 9 mmHg before surgery (76 eyes) to 15 ± 4 mmHg 5 years after surgery (56 eyes) (*t* = 7.683; *P* < 0.001). The mean IOP in the PXG group was not significantly different from that in the POAG group at 3 months, 6 months, and 1 year after surgery (*P*=0.193, 0.425, and 0.162). However, at 3 and 5 years after surgery, there was a statistically significant difference in the mean IOP between the groups (*P*=0.021 and 0.026).

The number of antiglaucoma medications in the PXG group decreased from 2.21 ± 1.01 before the surgery to 1.29 ± 1.20 at 5 years after the surgery, while that in the POAG group decreased from 1.89 ± 0.79 before the surgery to 0.84 ± 1.19 5 years after the surgery. The number of antiglaucoma medications used by the PXG group during the last follow-up was not significantly different from that of the POAG group (*P*=0.364, 0.726, 0.370, 0.106, and 0.088).

### 3.2. Surgical Success Rates

Among the 53 patients in the PXG group, 50 (94.3%), 42 (79.2%), and 35 (66.0%) completed the follow-up at 1, 3, and 5 years after trabeculectomy, respectively. The average follow-up period in the PXG group was 46.5 ± 20.5 months. Among the 76 patients in the POAG group, 68 (89.5%), 61 (80.3%), and 56 (73.7%) patients completed the follow-up at 1, 3, and 5 years after trabeculectomy, respectively, with an average follow-up period of 48.2 ± 20.9 months.

**Figure 1:**
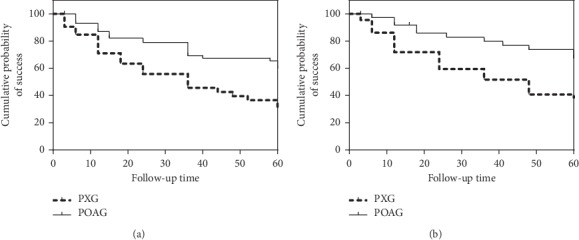
Kaplan–Meier survival analysis of complete (a) and qualified (b) success of trabeculectomy in patients according to criterion A in the PXG and POAG groups. Complete and qualified success rates were significantly different in the PXG and POAG groups (*P*=0.001, *P*=0.007, Mantel–Cox log-rank test, and criterion A).

**Figure 2:**
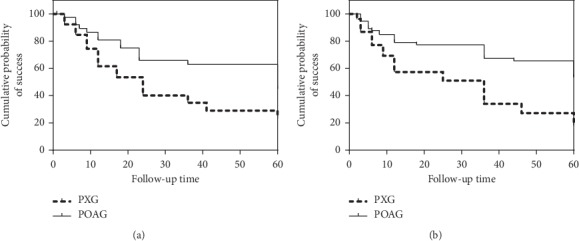
Kaplan–Meier survival analysis of complete (a) and qualified (b) success of trabeculectomy in patients according to criterion B in the PXG and POAG groups. Complete and qualified success rates were significantly different in the PXG and POAG groups (*P*=0.000, *P*=0.020, Mantel–Cox log-rank test, and criterion B).

**Figure 3:**
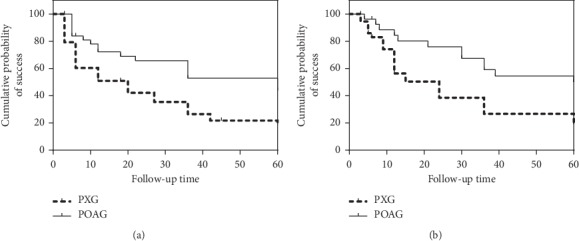
Kaplan–Meier survival analysis of complete (a) and qualified (b) success of trabeculectomy in patients according to criterion C in the PXG and POAG groups. Complete and qualified success rates were significantly different in the PXG and POAG groups (*P*=0.001, *P*=0.009, Mantel–Cox log-rank test, and criterion C).

A comparison of complete and qualified success rates of the surgery at different time points after trabeculectomy according to postoperative IOP control criteria A, B, and C between the two groups is shown in Figures 1–3 and Table 3. Complete success rates were significantly different in the PXG and POAG groups (*P*=0.001, *P*=0.000, *P*=0.001, Mantel–Cox log-rank test, and criteria A–C). Qualified success rates were significantly different in the PXG and POAG groups (*P*=0.007, *P*=0.020, *P*=0.009, Mantel–Cox log-rank test, and criteria A–C).

### 3.3. Postoperative Complications

Postoperative complications occurred in 11 (20.8%) eyes in the PXG group and in 12 (15.8%) eyes in the POAG group.

#### 3.3.1. Short-Term Postoperative Complications

Delayed anterior chamber formation occurred in 3 eyes in the PXG group, among which 1 eye spontaneously recovered without treatment and 2 eyes recovered after drug therapy. Hyphema occurred in 2 eyes, which recovered after anterior chamber flushing. Choroidal detachment occurred in 4 eyes, among which 3 recovered after drug treatment and 1 recovered after surgical treatment. Delayed anterior chamber formation occurred in 4 eyes in the POAG group, and hyphema occurred in 3 eyes, which all recovered spontaneously without treatment. Choroidal detachment occurred in 2 eyes and recovered after medical treatment.

#### 3.3.2. Long-Term Postoperative Complications

Filtration bleb leakage occurred in 2 (3.8%) eyes in the PXG group, but recovered after surgical treatment. Filtration bleb leakage occurred in 3 (3.9%) eyes in the POAG group, among which 1 eye recovered after injection of autologous blood around the filtration bleb and the other cases recovered after surgical treatment. No filtration bleb-related infection or other complications were noted in either group during the follow-up period (Table 4).

Additional secondary antiglaucoma surgery was carried out in 10 eyes in the PXG group (18.9%) and in 11 eyes in the POAG group (14.5%) during follow-up. In the PXG group, 5 repeat trabeculectomy and 5 combined Ahmed valve and phacoemulsification with intraocular lens implantation were conducted, and 6 repeat trabeculectomy and 5 combined Ahmed valve and phacoemulsification with intraocular lens implantation were conducted in the POAG group. No significant complications occurred in all cases.

## 4. Discussion

PXG develops from PEX and can seriously threaten vision. Its mechanisms primarily include mechanical blockage of the trabecular meshwork caused by intraocular exfoliation substances, dysfunction of the trabecular meshwork, and the coexistence of POAG. The combined action of these three factors leads to the development of PXG [11–13]. Due to the significant fluctuation of IOP, rapid progression of visual field damage, poor response to antiglaucoma medications, and low target IOP in this disease, surgical treatment is often required within a short period of time once the diagnosis is confirmed [6]. With the progression of PXG, the lamina cribrosa sclera will gradually become thinner. This change will render the eyeball more sensitive to changes in IOP. Therefore, setting individualized target IOP for different patients is a reliable method to evaluate the success of surgery. That is why we evaluated the success rate of surgery according to three different IOP criteria. The purpose of this study was to analyze the success rate of surgery for PXG and POAG in an Asian population.

In the past, there has been controversy regarding the results of comparative studies investigating the success rate of surgery for PXG and POAG. At 1 and 5 years after trabeculectomy, Lim and Cha [7] reported that the success rates in the PXG group were 84.4% and 19.9%, while the success rates in the POAG group were 82.3% and 64.7%, respectively. The complete success rate in the PXG group was lower than that in the POAG group, and there was no statistically significant difference in the qualified success rate between the two groups. The success rate in PXG patients was similar to that in POAG patients at 1 year after the surgery, but was lower than that in POAG patients at 2 years. They attributed the high failure rate of surgery in the PXG group to different definitions of the success rate, prolonged follow-up period, whether antimetabolic medications were used, the natural course of PXG, and ethnic differences. Pelitli et al. [14] reported that the success rates of surgery for PXG and POAG were similar. Ehrnrooth et al. [15] compared the success rates of surgery for PXG and POAG from 2 to 5 years after trabeculectomy and found that the complete success rate in patients with POAG was higher than that in those with PXG. The difference in the surgical success rate between those with PXG and POAG may have been caused by differences in sample selection and average age. In addition, surgical techniques, success criteria, and follow-up period were quite different in the study. Another important factor determining the success of trabeculectomy is the follow-up period—the longer the follow-up period, the lower the success rate.

Results of the present study demonstrated that according to criteria A, B, and C, there was no statistically significant difference in complete or qualified success rates between the PXG and POAG groups at 3 and 6 months after the surgery, suggesting that short-term success rates in the two groups are similar. According to criterion A, the complete success rates of the PXG group at 3 and 5 years after surgery were lower than those of the POAG group, suggesting that the long-term success rate of PXG patients was lower than that of POAG patients with the criterion of IOP ≤21 mmHg and under the condition of no medication after surgery; moreover, there was no significant difference in the surgical success rate between the two groups under the condition of medication control (i.e., ≤3 medications). For PXG patients, the target IOP criterion of 21 mmHg is relatively high [15, 16]; therefore, we adopted a more stringent criterion B. According to criterion B, the complete and qualified success rates of the PXG group were lower than those of the POAG group at 3 and 5 years after surgery, indicating that the long-term success rates of the PXG group were lower than those of the POAG group with the criterion of IOP ≤18 mmHg, and under the condition of no medication or medication control (i.e., ≤3 medications). Compared with criterion A, based on criterion B, the two groups of patients not only had statistically significant difference in the complete surgical success rate but also had statistically significant difference in the qualified success rate. Even if antiglaucoma medications were applied, the surgical success rate of PXG patients was still lower than that of POAG patients. As such, based on the higher criterion C, the complete and qualified success rates of the PXG group were lower than those of the POAG group at 1, 3, and 5 years after surgery, with a statistically significant difference, which indicates that the short-term success rate of the PXG group at 1 year after surgery was also low when the target IOP was set at a low level, and it was difficult to realize long-term low target IOP control.

It remains unclear why the qualified success rate of the PXG group gradually decreases with the extension of the follow-up period, given that the pathophysiological mechanism has not been fully clarified. Some studies have speculated that destruction of the blood-aqueous barrier after PXG trabeculectomy is an important cause of reduction in the success rate of the surgery. There are inflammatory manifestations, such as aqueous flare in PEX eyes, which indicates that the blood-aqueous barrier of PEX eyes has been destroyed [17]. The surgery will cause more serious damage to the original blood-aqueous barrier, which in turn will lead to a significant postoperative inflammatory reaction, fibrin exudation, formation of posterior synechia of the iris, which accelerates scarring of filtering blebs, and failure of the filtering function. Second, the destruction of the blood-aqueous barrier in PXG eyes after trabeculectomy is more serious than that in POAG, the levels of transforming growth factor beta (TGF-*β*) and endothelin-1 in aqueous humor increase, and iris angiopathy and systemic angiopathy are also involved, all of which will have an influence on the postoperative effects of trabeculectomy [18]. Third, exfoliation may promote scar formation in the filtering area. Iris angiopathy and destruction of the blood-aqueous barrier can cause postoperative cellulose-like exudation, and neovascularization of the iris can cause intraoperative or postoperative hyphema [19, 20]. In this study, one case of hyphema was found after PXG surgery.

In conclusion, trabeculectomy for PXG and POAG significantly reduced IOP. The short-term surgical success rates of both types of glaucoma were similar; however, the long-term success rate of PXG patients was significantly lower, and it was difficult to achieve long-term IOP control at a low target level.

## Figures and Tables

**Table 1:** General information for glaucoma patients in the two groups.

	PXG (*n* = 53)	POAG (*n* = 76)	*P*
Age (Y)	68.77 ± 7.37 (50–86)	68.62 ± 9.33 (51–88)	0.461^a^
Sex (M/F)	29/24	40/36	0.815^b^
Preoperative IOP (mmHg)	28 ± 9 (18–54)	26 ± 9 (16–52)	0.224^a^
Preoperative medications (*n*)	2.21 ± 1.01	1.89 ± 0.79	0.051^a^
MD (dB)	−24.59 ± 5.66	−22.96 ± 6.06	0.125^a^
Axial length (mm)	23.16 ± 0.96	23.01 ± 0.95	0.367^a^
MMC concentration (mg/mL)	0.31 ± 0.08	0.29 ± 0.08	0.243^a^
MMC time (min)	3.30 ± 1.14	3.10 ± 1.16	0.342^a^
Follow-up period (mo)	47.0 ± 19.9 (12∼63)	49.1 ± 19.5 (12∼65)	0.550^a^

^a^ *P* value calculated using the independent *t*-test; ^b^*P* value calculated using the chi-squared test; IOP, intraocular pressure; MD, mean defect; MMC, mitomycin C; PXG, pseudoexfoliation glaucoma; POAG, primary open-angle glaucoma.

**Table 2:** Comparison of IOP and medications between the two groups before and after trabeculectomy.

Time	Parameters	PXG		POAG		*P*
		*n*	IOP	*n*	IOP	
3 months	IOP (mmHg)	53	14 ± 3 (9–23)	76	13 ± 3 (8–23)	0.193
	Medications (*n*)	53	0.09 ± 0.30	76	0.05 ± 0.22	0.364
6 months	IOP (mmHg)	52	14 ± 3 (8–21)	73	14 ± 3 (8–22)	0.425
	Medications (*n*)	52	0.21 ± 0.54	73	0.18 ± 0.51	0.726
1 year	IOP (mmHg)	50	15 ± 3 (8–21)	68	14 ± 3 (8–24)	0.162
	Medications (*n*)	50	0.44 ± 0.79	68	0.31 ± 0.78	0.370
3 years	IOP (mmHg)	42	16 ± 4 (10–26)	61	15 ± 3 (9–23)	0.021^※^
	Medications (*n*)	42	0.90 ± 1.16	61	0.54 ± 1.03	0.106
5 years	IO (mmHg)	35	17 ± 4 (10–26)	56	15 ± 4 (9–24)	0.026^※^
	Medications (*n*)	35	1.29 ± 1.20	56	0.84 ± 1.19	0.088

*P* values were calculated using the independent *t*-test; ^※^significant. IOP, intraocular pressure; PXG, pseudoexfoliation glaucoma; POAG, primary open angle glaucoma.

**Table 3:** Comparison of surgical success rates between the two groups *n* (%).

Criteria	Time	Parameters	PXG		POAG		*χ* ^2^	*P*
			*n*	Success rates	*n*	Success rates		
Criterion A (<21 mmHg)	3 months	Complete	53	47 (89)	76	70 (92)	0.123	0.726
		Qualified	53	49 (93)	76	72 (95)	0.025	0.874
	6 months	Complete	52	44 (85)	73	64 (88)	0.241	0.623
		Qualified	52	47 (90)	73	67 (92)	0.000	1.000
	1 year	Complete	50	36 (72)	68	55 (81)	1.288	0.256
		Qualified	50	41 (82)	68	59 (87)	0.506	0.477
	3 years	Complete	42	23 (55)	61	45 (74)	4.006	0.045^※^
		Qualified	42	29 (69)	61	47 (77)	0.823	0.364
	5 years	Complete	35	14 (40)	56	35 (63)	4.388	0.036^※^
		Qualified	35	21 (60)	56	42 (75)	2.275	0.131

Criterion B (<18 mmHg)	3 months	Complete	53	45 (85)	76	68 (90)	0.600	0.439
		Qualified	53	46 (87)	76	69 (91)	0.516	0.473
	6 months	Complete	52	40 (77)	73	58 (80)	0.115	0.735
		Qualified	52	42 (81)	73	61 (84)	0.163	0.686
	1 year	Complete	50	34 (68)	68	52 (77)	1.046	0.306
		Qualified	50	37 (74)	68	56 (82)	1.204	0.273
	3 years	Complete	42	18 (43)	61	39 (64)	4.471	0.034^※^
		Qualified	42	23 (55)	61	46 (75)	4.796	0.029^※^
	5 years	Complete	35	10 (29)	56	31 (55)	6.242	0.012^※^
		Qualified	35	16 (46)	56	38 (68)	4.377	0.036^※^

Criterion C (<15 mmHg)	3 months	Complete	53	40 (76)	76	61 (80)	0.422	0.516
		Qualified	53	41 (77)	76	62 (82)	0.346	0.557
	6 months	Complete	52	33 (64)	73	57 (78)	3.220	0.073
		Qualified	52	35 (67)	73	59 (81)	2.974	0.085
	1 year	Complete	50	28 (56)	68	50 (74)	3.951	0.047^※^
		Qualified	50	30 (60)	68	53 (78)	4.445	0.035^※^
	3 years	Complete	42	14 (33)	61	34 (56)	5.017	0.025^※^
		Qualified	42	18 (43)	61	39 (64)	4.471	0.034^※^
	5 years	Complete	35	8 (23)	56	29 (52)	7.471	0.006^※^
		Qualified	35	13 (37)	56	33 (59)	4.090	0.043^※^

*P* values were calculated using the chi-squared test. ^※^*P* < 0.05. IOP, intraocular pressure; PXG, pseudoexfoliation glaucoma; POAG, primary open-angle glaucoma.

**Table 4:** Comparison of postoperative complications between the two groups *n* (%).

Complications	PXG (*n* = 53)	POAG (*n* = 76)	*P*
Shallow anterior chamber	3 (5.7)	4 (5.3)	1.000
Hyphema	2 (3.8)	3 (3.9)	1.000
Choroidal detachment	4 (7.5)	2 (2.6)	0.192
Filtering bleb leaking	2 (3.8)	3 (3.9)	1.000
Total	11	12	0.469

*P* values were calculated using the Fisher exact test. PXG, pseudoexfoliation glaucoma; POAG, primary open angle glaucoma.

## Data Availability

The research data used to support the findings of this study are available from the corresponding author upon request.
